# Epithelial ovarian carcinoma diagnosis by desorption electrospray ionization mass spectrometry imaging

**DOI:** 10.1038/srep39219

**Published:** 2016-12-15

**Authors:** Maria Luisa Dória, James S. McKenzie, Anna Mroz, David L. Phelps, Abigail Speller, Francesca Rosini, Nicole Strittmatter, Ottmar Golf, Kirill Veselkov, Robert Brown, Sadaf Ghaem-Maghami, Zoltan Takats

**Affiliations:** 1 Department of Surgery and Cancer, Faculty of Medicine, Imperial College London, London, United Kingdom

## Abstract

Ovarian cancer is highly prevalent among European women, and is the leading cause of gynaecological cancer death. Current histopathological diagnoses of tumour severity are based on interpretation of, for example, immunohistochemical staining. Desorption electrospray mass spectrometry imaging (DESI-MSI) generates spatially resolved metabolic profiles of tissues and supports an objective investigation of tumour biology. In this study, various ovarian tissue types were analysed by DESI-MSI and co-registered with their corresponding haematoxylin and eosin (H&E) stained images. The mass spectral data reveal tissue type-dependent lipid profiles which are consistent across the n = 110 samples (n = 107 patients) used in this study. Multivariate statistical methods were used to classify samples and identify molecular features discriminating between tissue types. Three main groups of samples (epithelial ovarian carcinoma, borderline ovarian tumours, normal ovarian stroma) were compared as were the carcinoma histotypes (serous, endometrioid, clear cell). Classification rates >84% were achieved for all analyses, and variables differing statistically between groups were determined and putatively identified. The changes noted in various lipid types help to provide a context in terms of tumour biochemistry. The classification of unseen samples demonstrates the capability of DESI-MSI to characterise ovarian samples and to overcome existing limitations in classical histopathology.

Ovarian cancer is the fifth most common cancer in Europe among women and the most common cause of gynaecological cancer death^1^. Investigation of ovarian cancer is primarily based on medical imaging with ultrasound and computed tomography (CT) and serological biomarkers, including carbohydrate antigen-125 (CA-125). Once the presence of a tumour has been confirmed, its histological diagnosis must be determined after surgery as imaging and serological markers are not sufficiently specific for discriminating benign, borderline or malignant tumours. Histological information is then provided by a histopathologist and complemented by further diagnostic tests including immunohistochemistry^2^. Histological information is based on human interpretation and is, therefore, prone to inter-observer variability and, equally, diagnostic variability^3^,^4^.

Mass spectrometry (MS) is an essential tool for proteomic biomarker discovery^5^ and it is also increasingly used to investigate lipids and other small molecules as markers of ovarian cancer^5^. Its application in the field of lipidomics allows the identification, characterisation, and quantitation of all known lipid species.

Lipid metabolism undergoes a drastic change in cancer where the metabolism converges to lipid synthesis. More precisely there is a shift from lipid uptake to *de novo* lipogenesis^6^, which has an impact on the fatty acid (FA)^6^ and lipid compositions of the entire cell. The mechanism of cancer progression’s impact on lipid biochemistry is still not properly understood^7^,^8^.

Mass spectrometry imaging (MSI) has been gaining importance due to its ability to provide not only the metabolic profile of a sample but also spatial information. This can provide the identity and spatial distribution of a diverse range of biomolecules, such as peptides, drugs, and lipids^9^.

A few matrix-assisted laser desorption ionisation (MALDI) imaging mass spectrometry studies have already described MSI analyses of ovarian cancer^10^,^11^, however, most of these studies followed a protein biomarker discovery approach, rather than using lipidomic profiles and multivariate statistical tools.

Amongst MSI techniques, desorption electrospray ionisation (DESI) MSI is increasingly used for histological studies because it does not require the use of ionisation matrices. Consequently there are no matrix-related sample preparation problems, including variance or interference from matrix peaks in the MS spectrum and fewer sample preparation steps are needed, making this technique simpler to use and less prone to errors^12^. In DESI, electrically charged solvent micro-droplets are directed onto a sample’s surface and the impact of these primary charged particles on the sample’s surface produces secondary ions, from the sample, which are analysed with a conventional mass spectrometer^13^.

Based on their lipid profiles, DESI-MSI allows the acquisition of tissue type-specific ion patterns and enables robust tissue recognition and identification. As a result, DESI has been widely used for chemical imaging and diagnoses of tissue samples. The idea of DESI-MSI as a cancer diagnostic tool and profiling of different tissue types was first shown in human liver adenocarcinoma^14^. In addition to liver, DESI-MSI has also been proven as an effective technique for a variety of different cancer types, such as brain^15^, breast^16^, prostate^17^, bladder^18^, stomach^19^, colon and rectum cancer tissues^12^ and extensively reviewed this year^20^,^21^. Ovarian cancer was recently evaluated in a mouse model reproducing the clinical nature of human high grade serous ovarian carcinoma^22^. However, analysis by DESI-MSI hasn’t previously been reported in human ovarian cancer.

The ability to directly correlate MSI with histological features under the microscope provides topographically localised biochemical information which could potentially supplement conventional histological classification systems and provide more relevant clinical information, which could potentially impact clinical decisions.

In this study, DESI-MSI was used to profile the lipidome of different epithelial ovarian carcinomas (EOC) and borderline ovarian tumours together with normal ovarian stroma and normal fallopian tube. This approach allowed the investigation of the lipid biology and lipid distribution in these gynaecological tissues. This technology may have the potential to provide faster and user-independent tissue diagnoses, using the tissues’ spatial lipidomic distributions.

## Results

**Figure 1:**
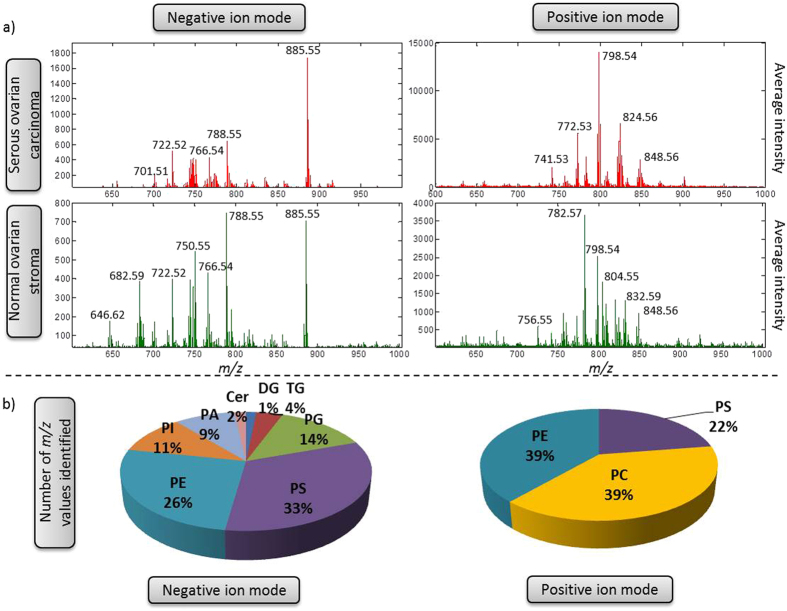
(**a**) Average spectra of two different samples, serous carcinoma from ovary and normal stroma from apparently normal ovary analysed in positive ion mode and negative ion mode. (**b**) Percentage of lipid species classes identified based on online databases.

DESI-MSI can provide the lipidomic profile and this profile can be used to determine tumour type as well as tissue type. Figure 1 shows the average spectrum from a section of normal ovary and from serous ovarian carcinoma; both in negative and positive ion modes. The most abundant classes of phospholipids in negative ion mode for the samples were phosphatidylserine (PS) and phosphatidylethanolamine (PE), followed by phosphatidylglycerol (PG) and phosphatidylinositol (PI). In positive ion mode the spectra were dominated by phosphatidylcholine (PC) and PE. Negative ion mode covers a wider range of phospholipid classes but is complemented by positive ion mode when PCs are more commonly seen. For the different polarities analysed by DESI-MSI, adjacent sections were used for both positive and negative ion mode acquisitions.

### DESI and spatial tissue identification

The spatially resolved nature of DESI-MSI permits identification and prediction of different tissue types within, what may be considered to be, macroscopically homogenous tissue. Using the histologically annotated pixels in each tissue section, a sample’s remaining pixels can be classified using supervised multivariate analyses.

**Figure 2:**
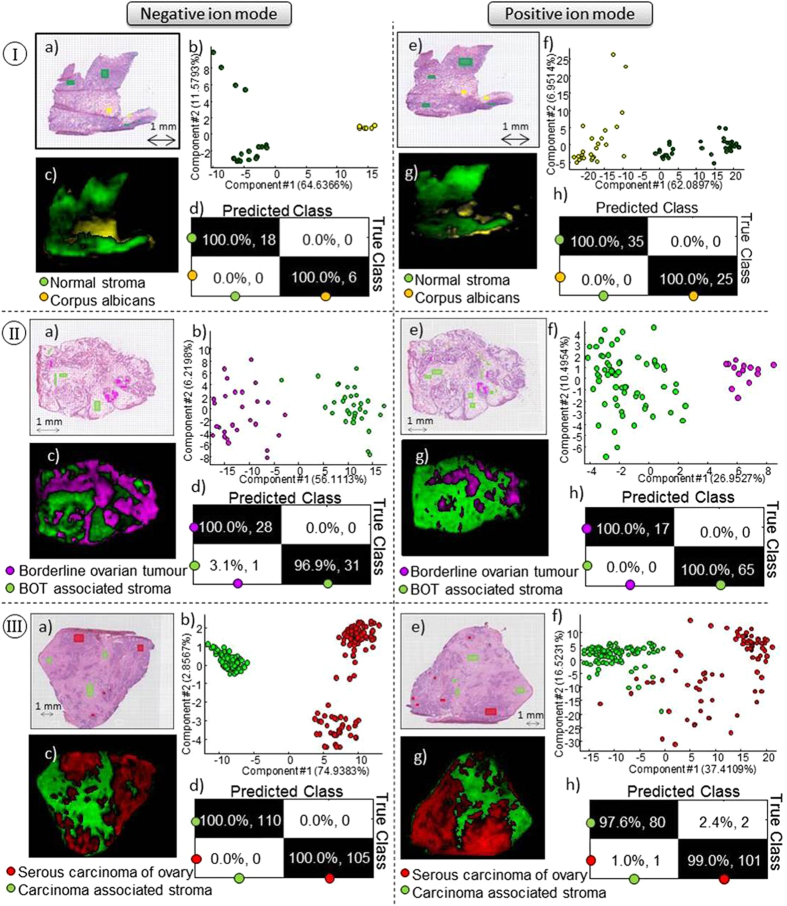
(**I**) Normal ovary with two different tissue types, normal stroma (green) and corpus albicans (yellow). (**II**) Serous borderline tumour and (**III**) Serous carcinoma with two tissue types in each sample, stroma (green) and tumour tissue (red and pink). Data acquired in negative ion mode and positive ion mode. (a) and (e) Optical images and (b) and (f) PC analyses of selected regions of interest. (c) and (g) predicted RMMC components with (d) and (h) leave-one-out cross validation results.

Two distinct tissue types were identified by the histopathologist; connective tissue and corpus albicans (Fig. 2Ia and e). The PCA scores plots in Fig. 2Ib and f in both negative ion mode and positive ion mode demonstrate that there is a clear difference between the two lipid profiles. The pixel predictions (Fig. 2Ib and h) are clearly in line with the observed histology in the H&E image, which is more obvious in negative ion mode (Fig. 2Ia–d) than in positive ion mode (Fig. 2Ie–h).

In addition to normal ovarian tissue samples, sections containing three histological types of EOC were also analysed, as well as borderline serous ovarian tumour samples. Figure 2II and III as well as Supplementary Figure 2 shows the histology, PCA scores and sample prediction for sections containing each of these tissue types analysed in negative and positive ion mode. Both positive and negative ion modes demonstrate robust data to build strong models capable of discriminating the different tissue types, based on the different lipid composition of the tissues. All further work was performed on negative ion mode data due to the prevalence of observed lipid classes.

### Comparison of multiple samples

The results seen in Fig. 2 demonstrate the ability of DESI-MSI to provide spatially resolved mass spectra and thus specific lipidomic profiles for different tissue types. Further analyses focussed on global lipidomic trends across multiple samples.

**Figure 3:**
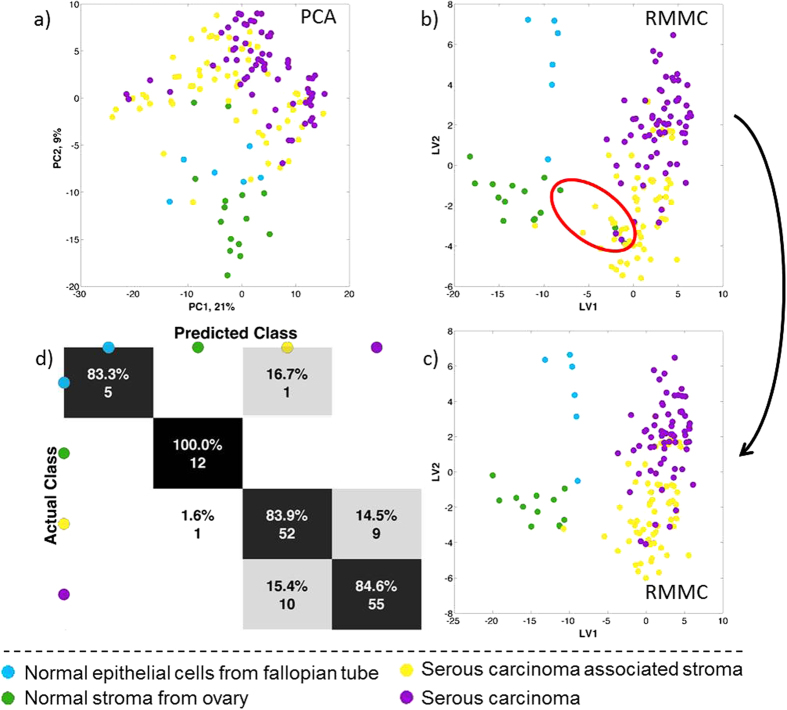
Statistical analysis of DESI-MSI spectrum between 600–1000 Da from serous carcinoma samples with two tissue types (stroma and carcinoma) together with normal stroma from normal ovary and normal epithelium from fallopian tube. (**a**) Principal component analysis (PCA) and (**b**) Maximum margin criteria analysis (RMMC) and (**c**) RMMC analysis after excluding the 3 normal appearance stroma from cancer patients with respective (**d**) leave one patient out cross validation using Mahalanobis as a classifier.

Figure 3 shows the results of analyses performed using 4 distinct tissue types: serous carcinoma, carcinoma associated-stroma, normal fallopian tube and normal ovary. For these analyses the regions of interest were selected by a qualified histopathologist from regions where only a single tissue type was identified. Histopathologist-annotated spectra across the various samples were peak matched, normalised and log transformed prior to multivariate analysis as previously described.

Whilst there is a noticeable degree of overlap in the PCA scores plots (Fig. 3a) of the four tissue types, the second component does show a trend separating the two normal controls (fallopian tube and ovary) from the carcinoma-associated stroma and serous carcinoma. Thus, in spite of the sample and patient variability, each tissue type has a consistent lipid profile across the various tissue sections.

Three samples, circled in Fig. 3b, were classified as normal ovary by the histopathologist but classified by RMMC as carcinoma-associated stroma. Interestingly, these three samples are from patients with ovarian cancer but distant from the histopathological sampling area. These misclassified samples were removed from all subsequent analyses (reducing the normal ovary class from 15 to 12 samples) once they were confirmed by histology to be neither normal ovarian tissue nor actual tumour tissue. Upon removing these samples, RMMC analysis (Fig. 3c) was used to classify the remaining samples and assessed by means of a leave-one-patient-out cross validation scheme. Figure 3d shows the confusion matrix for tissue type classification with an overall accuracy of 85%. The first latent variable seen in Fig. 3c discriminates the two normal tissue types from the carcinoma-associated tissue, whilst the second latent variable shows a noticeable separation between the two normal groups and also a separation between serous carcinoma and all stroma samples.

### Lipidomic profile of each tissue type

The previous multivariate analyses demonstrate a difference between the various tissue types, for which differences in the lipid profiles are responsible. In order to determine which phospho- and sphingo-lipid species were driving the differentiation seen in Fig. 3c, average spectra and component weights from RMMC were compared (Supplementary Figures 3 and 4).

The average spectra (Supplementary Figure 3) reveal noticeable differences between groups such as, for example, the peaks at *m/z* 788.55 and 682.59 showing higher abundances in normal tissues compared to the normal stroma and serous carcinoma tissue types (shown also in Fig. 1). The RMMC component weights for latent variables 1 and 2 are shown in Supplementary Figure 4 and Supplementary Table 1. They reveal that five distinct lipid classes are responsible for the separation between the normal tissues and stroma/serous carcinoma tissues: phosphatidic acids (PA), PS, PE, PG and ceramides (Cer).

Ceramides contribute to the separation of normal from carcinoma whilst this is reversed for PAs, showing elevated PAs in carcinoma compared to normal tissues. PEs are not uniformly discriminatory as, for example, PE(38:6) and PE(40:3) corresponding to the *m/z* values of 762.51 and 796.59 respectively, have positive values in LV1, increasing in carcinoma and carcinoma-associated stroma compared to normal tissue. In contrast PE(34:0) and PS(34:1), corresponding to the *m/z* values of 718.54 and 760.52 respectively, have large negative weights in LV2 meaning they are decreased in cancer-associated stroma compared to cancer tissue.

**Figure 4:**
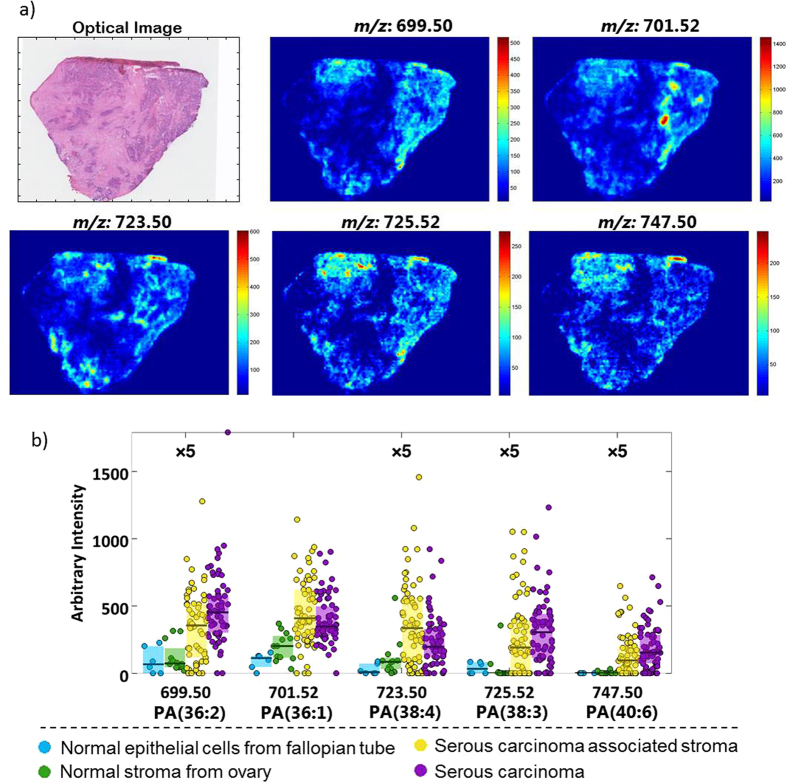
Characterization of phosphatidic acid class (PA) in ovarian cancer. (**a**) Ion images of 5 different PA species in a serous ovarian carcinoma with (**b**) Box plots of the same lipid species.

The variables shown in Supplementary Table 2 and used for Fig. 4 all have absolute mean log_2_ fold changes (MFC) equal to or greater than 1, FDR-corrected q-values < 0.05 and intensities >10. Using the high accuracy *m*/*z* values and their isotopic patterns, compounds were tentatively identified as described in the Methods.

One of the most consistent changes related to lipid composition is the PA class, where the most significant peaks are higher in the tumour tissue and its associated stroma compared to both controls of normal ovary and fallopian tube. MFC is significantly higher in the serous carcinoma versus normal ovary compared to carcinoma-associated stroma versus normal ovary. One exception to this is PA(38:4) which is higher in stroma than it is in tumour. Figure 4a illustrates the distribution of the five different PAs identified, where the intensities in tumour tissue are always greater than in carcinoma-associated stromal tissue.

The PS class also presents noteworthy changes, as short chain PSs are higher in normal ovary (PS(34:1) and PS(36:1)) but long chain PSs behave in the opposite manner, presenting a high MFC when comparing tumour with both control groups but not when comparing carcinoma-associated stroma with the control samples. The PE class presents different changes for the different peaks identified; plasmalogen species, such as PE(P-36:2) and PE(P-38:3), show higher intensities in tumour tissue compared to the carcinoma-associated stroma and both control tissues. PE(36:2) and PE(38:3) are also elevated in the tumour tissue but show a gradual change from normal ovary to carcinoma-associated stroma to tumour. The final class of identified compounds is phosphatidylinositol (PI), with a slight increase in carcinoma, except the plasmalogens, which appear to be increased in normal ovary with a MFC of approximately two.

### Ovarian cancer diagnosis

As with all cancers, accurate diagnosis is crucial as a patient’s treatment plan is often influenced by the histopathological tissue analysis. Histopathology is currently the ‘gold standard’ procedure for tissue diagnosis and has shown that EOC comprises 90% of ovarian carcinomas.

In order to determine lipidomic differences between cancer and normal tissues, an initial statistical analysis for ovarian carcinoma diagnosis was performed by comparing all EOC (serous, endometrioid and clear cell) spectra against normal ovarian tissue. Additionally, epithelial tissue from fallopian tubes was included as another control group as the three EOCs have their origin in epithelial cells (Supplementary Figure 6).

**Figure 5:**
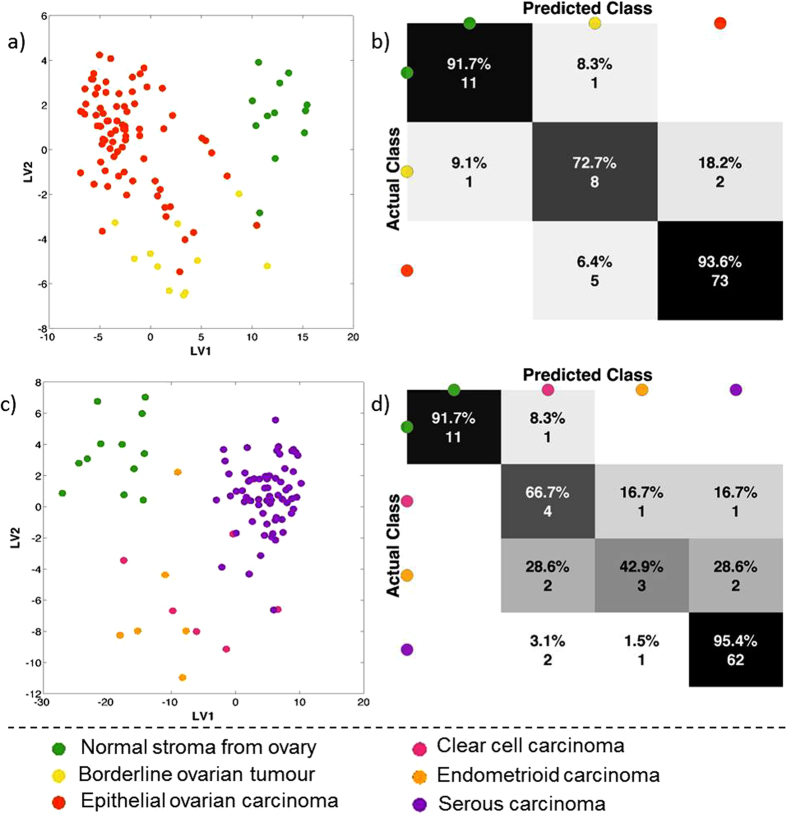
Statistical analysis of DESI MS spectra between 600–1000 Da from two different perspectives: all carcinomas together, borderline ovarian tumours and normal stroma from normal ovary (**a** and **b**) and from the different carcinomas as serous, endometrioid and clear cell carcinomas together with normal stroma from normal ovary (**c** and **d**). (**a** and **c**) shows maximum margin criteria analysis (RMMC) cross validated and (**b**) and (**d**) shows leave-one-patient-out cross validation results.

Borderline ovarian tumour tissue is often difficult to discriminate from carcinoma as they share many similar characteristics. As such, it would be beneficial to develop robust tools to accurately identify borderline tumours by characterising their shifting metabolism towards carcinoma. Multivariate analysis of normal, borderline and carcinoma samples was performed, and the results are shown in Fig. 5a and b.

Supervised analysis in Fig. 5a shows a much clearer continuum from normal to carcinoma, through borderline, tissue types. The confusion matrix in Fig. 5b shows an overall accuracy of 91%, with none of the misclassifications being between the carcinoma and normal pixels.

Borderline tumours can be difficult to diagnose, which may explain the borderline tumour samples being misclassified as carcinoma and *vice versa*^23^. The supervised classification results point to there being differences in the lipidomic profiles across the continuum of these three tissue groups. This represents altered metabolism in these tissues and may give insight into the underlying biological mechanisms responsible for the pathogenesis of malignancy in ovarian tissue.

Serous carcinomas are the most common type of EOC, followed by endometrioid, clear cell and mucinous carcinomas, which explains the lower sample number in the non-serous groups (Table 1). Multivariate comparisons of three carcinoma groups (endometrioid, clear cell and serous carcinomas) are shown in Fig. 5c and d along with normal ovarian tissue. From a lipidomic perspective, serous carcinoma is highly distinct from both clear cell and endometrioid carcinomas. However, these latter two are essentially indistinguishable from each other when comparing their lipidomic profiles.

### Pixel-wise classification of a blind sample

DESI-MSI has been demonstrated to be capable of predicting pixels within a sample and across multiple samples employing leave-one-out cross-validation scheme. The ultimate application of DESI-MSI in a clinical setting would be the prediction of a totally unseen sample, as it may provide a comprehensive histological diagnosis more quickly and objectively than could be provided by a histopathologist. Thus DESI-MSI could be a powerful tool for the identification and diagnosis of cancer.

The prediction of an entirely independent sample was performed using a training set derived from all previously analysed tissue samples and encompassing serous ovarian carcinoma, carcinoma-associated stroma and normal ovarian tissue. The pixel size of this analysed blind sample was also 100 μm × 100 μm. An iterative one-class-against-all RMMC model was used to determine the probability of each test-set pixel belonging to each of the classes in the training set. Pixels with a single probability *p* > 0.99 were deemed to be unambiguously classified. Those with *p* < 0.99 were unclassified as were those with multiple probabilities *p* > 0.99.

**Figure 6:**
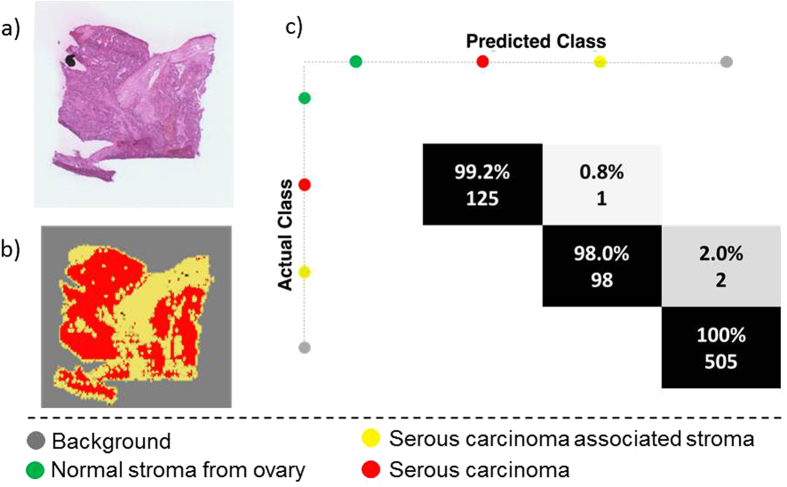
Prediction of an independent sample using as training set the previous model (serous ovarian carcinoma). (**a**) Optical image of the independent sample, (**b**) predicted image and (**c**) confusion matrix for classification of histopathologist-annotated tissue regions of interest.

A histopathologist had previously annotated regions of interest in this blind sample, and the classification of these regions of interest (pixels) is shown in the confusion matrix in Fig. 6c, where an overall accuracy of 99.6% was achieved. More examples are provided in Supplementary Figure 8, which demonstrates the wide capacity of the data to predict unseen samples. It is envisioned that the acquisition and inclusion of further samples will increase the predictive capacity of the model and result in enhanced classification performance.

## Discussion

The application of DESI-MSI in the characterisation of different cancers demonstrates the potential of this technique as a diagnostic tool, complementing and overcoming some of the major issues in the current histopathological workflows. Notably, we can achieve a robust model to perform pixel-wise classification of a completely independent blind sample (Fig. 6).

The applications of DESI-MSI are vast and it has previously been shown to accurately determine tissue type and to provide further strong evidence that DESI-MSI can serve as a powerful tool to characterise and identify the lipid profile of different tissue types in cancer along with the application of supervised and unsupervised statistical techniques. Gastric cancer samples have been analysed by various groups with different approaches, such as with least absolute shrinkage and selection operator (LASSO)^24^ where several lipids, such as cardiolipins and sphingomyelins, were identified as being specific across various tissue types (gastric cancer, normal epithelial, and stromal gastric tissue). The analysis of metastatic lymph nodes^19^, also from gastric cancer, used PCA and RMMC, which completely distinguished between the tissue types found in the samples, whilst also identifying various differentiating lipids. For the analysis of breast cancer samples^16^, RMMC analysis was also employed to characterise the different cancer types according to their hormone receptor status.

Whilst other tissues have been well studied by DESI-MSI, ovarian cancer, however, has not been widely analysed in terms of tissue characterisation and lipid analysis. A mouse model study has previously been presented which mimics human high grade serous ovarian carcinoma^22^. Some of the tumour/healthy differentiating lipid species are in agreement with those identified in our study, such as certain ceramides, PIs, PEs and PSs.

Studies such as these generate a tissue classification model, which enables the study of variation in lipidomic profiles across tissue types. The spatial resolution of DESI-MSI makes it possible to distinguish between the two tissue types that are routinely found in ovarian samples (stroma and tumour tissue), and also to assess the differences in their lipidomic profiles within the same sample. In our study, we compared the epithelial tissue from normal fallopian tube and the stroma from normal ovary. Interestingly, in our supervised analysis, the second latent variable shows that epithelial cells from fallopian tube are closer to serous cancer cells than to normal ovarian stroma, which may reflect the common ontogenesis between these epithelial cells. The cellular origin of EOC has been long debated as these tumours are derived from mullerian ducts and are therefore mullerian derived epithelial tissue rather than mesodermal epithelium from which the ovaries develop^25^. The recent literature therefore suggests that EOC is not derived from the ovarian surface epithelium (mesodermal), as originally thought, but derives from mullerian-type tissue (cervix, endometrium, and fallopian tubes)^26^. Unfortunately, in our cohort we were unable to collect ovarian surface epithelium to evaluate which epithelial tissue would be most similar to the tumour in order to support the fallopian tube origin theory described above.

Another very interesting point is the analysis of the three different EOC histological types, where clear cell and endometrioid carcinomas are not distinguishable from each other but completely separate from serous carcinoma. This could stem from the fact that these two cancers usually coexist in the tumour and also in part due to the low sample numbers in this study. Furthermore, recent studies have suggested that clear cell and endometrioid-type carcinomas of the ovary are likely to arise from endometriosis^27^ and this may explain why metabolic changes in these tissue types seem to be similar. There are some studies about the histogenesis of endometriosis and how it differs to explain the development of endometrioid and clear cell carcinomas^28^. However, this dichotomy is far from being completely understood.

Unfortunately, in-depth analysis of similarities and differences between endometrioid and clear cell cancer was limited in this study by the small sample size, which reflects the small number of patient samples available in the study.

Most mass spectrometry-based studies performed on ovarian cancer samples are based on comprehensive extraction and chromatographic analysis with GC-MS or LC-MS. Some studies using LC-MS have demonstrated the potential of the phospholipid profile for characterization and diagnosis of ovarian cancer^29^,^30^. Stuphen *et al*.^29^ and Shan *et al*.^30^ described lyso PAs as the class which was consistently increased in preoperative cancer patients. Interestingly, in our study, the PA class is always increased in serous cancer tissue compared to stroma and control ovaries. PA(40:6), for example, is barely detected in the control ovaries and epithelial tissue from fallopian tube, but shows a high abundance in both tumour-related tissues.

PA is a critical and essential phospholipid class for membrane phospholipid biosynthesis. The conversion of PA to diacylglycerol (DAG) and cytidine diphosphate diacylglycerol (CDP-DAG) are the two main arms of the phospholipid pathway, where DAG can precede PC and PE (Kennedy pathway), and CDP-DAG can precede PG, PI and PS (CDP-DAG pathway). As such, for the cells to proliferate at the astonishing rate observed in cancer, the membrane phospholipids have to increase accordingly^31^.

Furthermore, PAs also serve as critical lipid second messengers that regulate several proteins implicated in the control of cell cycle progression, cell growth and survival. For example, it is clear that PAs are essential for protein kinase mTOR (mammalian/mechanistic target of rapamycin) activity and mTOR activity is required for progression from G1 into S-phase, indicating that PAs, via input from mTOR, are required for cell cycle progression^32^,^33^. PAs were first demonstrated to be involved in the regulation of mTOR in 2001^32^, showing that exogenously added PAs stimulated the activation of the mTOR substrate S6 kinase and phosphorylation of another mTOR substrate ‘eukaryotic initiation factor 4E binding protein-1’ (4E-BP1) in human embryonic kidney (HEK293) cells.

Due to its link to survival signals, mTOR has been implicated in several human cancers^34^. PAs have a direct role stimulating mTOR, competing with rapamycin’s action, making it a potential target for therapy.

Consistent with this emerging role for PA in regulating cell proliferation, elevated expression and/or activity of enzymes that generate PA is commonly observed in human cancer. There are three major sources of PA: phospholipase D (PLD), diacylglycerol kinase (DGK), and lysophosphatidic acid acyltransferase (LPAAT). The LPAAT pathway is integral in *de novo* membrane phospholipid biosynthesis, whereas the PLD and DGK pathways are activated in response to growth factors and stress. The most described enzyme related to PA and cancer is PLD^35^.

Other phospholipids in serum have been shown to be important for an accurate diagnosis of ovarian cancer^30^. In addition to lyso PAs, both PEs and lyso PCs also play an important role in ovarian cancer diagnosis. In our study, despite being tissue rather than serum, PE(P-36:2) and PE(P-38:3) also show higher intensities in tumour tissue compared to the carcinoma-associated stroma and both control tissues. PE(36:2) and PE(38:3) are also elevated in the tumour tissue with a gradual change from normal ovary to carcinoma-associated stroma to tumour. This may demonstrate that PE species could play an important role in ovarian cancer diagnosis which may permit further insights in cancer biochemistry.

Another hallmark of cancer, functionally related to the glycolytic pathway, is increased *de novo* fatty acid (FA) synthesis^36^, evidenced by increase 0 fatty acid synthetase (FASN) which is well described in the literature^6^,^37^. These metabolic changes are functionally related to the glycolytic pathway and the well-known Warburg effect^38^. Acetyl-CoA and subsequently citrate are influenced by the increase of ATP citrate lyase (ACLY) to augment *de novo* fatty acid synthesis and give a quick response to cell growth and proliferation.

In this study, although the main focus was on the phospholipid and sphingolipid profile, we could observe changes in the fatty acid content of the different phospholipids identified. Our study evidences a shift in the composition of fatty acids from short chain fatty acids (SCFA) to long chain fatty acids (LCFA) in two of the most abundant classes of phospholipids detected, PS and PE. Focusing on the PS class, all identified compounds with a positive log_2_ mean fold change possess 40 or more carbons in their fatty acid chains and more than four double bonds. On the other hand, the compounds with a negative fold change all possess less than 40 carbons in their fatty acid chains or less than four double bonds, suggesting a shift of fatty acid composition/metabolism.

In fact, elongation of fatty acids in cancer is gaining importance. The increase in *de novo* synthesis also implies overexpression of elongation and desaturation processes in *de novo* lipogenesis. The overexpression of ELOVL1 (as described in the human protein atlas^39^) is strong evidence of further fatty acid synthesis for cancer metabolism and consistent with our results.

The focus on lipidomics and MS-based analysis in cancer metabolism is growing rapidly. However, findings in the literature are contradictory regarding the relative abundance of glycerolipids and sphingolipids between cancer and control groups^40^. This shows that we are still far from fully understanding cancer metabolism. In conclusion, this study is the first to apply DESI-MSI to ovarian cancer samples with a view to diagnostic applications. Based on their lipidomic profile, different EOC types, as well as different tissue types including tumour tissue and the surrounding stroma can be characterised and differentiated by DESI-MSI. This technique shows great potential for tissue characterisation and assignment, complementing classical histological diagnostic pathways.

## Methods

### Clinical patient data

The collection of all samples for this study was approved by the institutional review board at Imperial College Healthcare National Health Service Trust (Tissue Bank sub-collection number GYN/HG/12/060). All patients provided informed consent to the use of their samples in this study, and all methods were performed according to institutional and ethical guidelines. Patients undergoing gynaecological surgery were recruited and 89 tissue samples were taken from a range of borderline and EOC tumours. Normal control samples included normal ovaries (15) and fallopian tubes (6). All tissue samples were stored at −80 °C after collection (Table 1).

### Sample preparation and mass spectrometry analysis

Each sample was cryosectioned to a thickness of 12 μm, mounted onto a glass slide and stored at −80 °C prior to DESI-MSI measurement. After DESI-MSI measurement, tissue sections were stained with hematoxylin and eosin (H&E) and underwent histological examination by a histopathologist.

For DESI-MSI analysis, tissue sections were thawed under nitrogen flow. DESI-MSI was carried out using a home-built DESI ion source coupled to an Exactive Fourier transform Orbitrap mass spectrometer (Thermo Scientific, Bremen, Germany). The DESI-MSI was operated at a spatial resolution of 100 μm (injection time of 1000 ms) using nitrogen pressure of 7.0 bar, sprayer voltage of 4.5 kV, capillary voltage 50 V, capillary temperature of 250 °C, tube lens voltage of 150 V, and skimmer voltage of 40 V and a solvent mixture of methanol and water in a ratio 95:5 with a flow rate of 1.5 μL/min. The mass analysis was carried out in negative and positive ion modes over the mass range *m*/*z* 150–2000 at a nominal mass resolution of 100,000 and a mass accuracy of ±2 ppm. Each sample had between 40 and 150 mm^2^ acquired with a speed rate of 100 μm/s. The sample run order was randomly determined such that similar samples were not run consecutively.

### Sample processing

Following DESI-MSI acquisition, raw mass spectrometric images were converted to imzML format^41^ using imzML Converter (version 1.0.5) and imported into MATLAB (R2014a) for pre-processing and analysis^42^.

The tissue types present in each sample were determined following examination of the H&E-stained image by an independent histopathologist. Each histological image was co-registered to the DESI-MS image in order to facilitate histological annotation. A region of interest within both the H&E optical image and matched ion-image was selected by the histopathologist and all pixels/spectra within it were assigned a histological type and used in subsequent multivariate analyses.

The spectral profiles within the *m/z* region of 600–1000 were aligned using an in-house developed peak-matching procedure subject to a maximum peak shift of 8 ppm. Mass spectra from the same tissue and the same section were averaged (mean) and total ion count (TIC) normalised.

These averaged spectral profiles were analysed by principal components analysis (PCA) and recursive maximum margin criterion linear discriminants analysis (RMMC)^42^. RMMC was used for supervised discrimination and robust classification of spectra was performed using a leave-one-patient-out cross-validation scheme. In such a scheme, a supervised model is generated with data from all but the left-out patient. This model is then used to predict the spectra from the omitted patient, and the classifications are compared to the histopathologist’s annotations. These results are presented as confusion matrices showing the actual and predicted classifications of each spectral profile. Discriminating spectral variables were determined by inspection of the component weights in conjunction with false discovery rate-corrected univariate statistics (analysis of variance, ANOVA).

The variables that were found to discriminate significantly between tissue types were subsequently subjected to spectral annotation. This was performed using exact *m*/*z* values and isotopic distributions via database searches [Metlin^43^, Lipid maps^44^, HMDB^45^].

## Additional Information

**How to cite this article**: Dória, M. L. *et al*. Epithelial ovarian carcinoma diagnosis by desorption electrospray ionization mass spectrometry imaging. *Sci. Rep.* **6**, 39219; doi: 10.1038/srep39219 (2016).

**Publisher's note:** Springer Nature remains neutral with regard to jurisdictional claims in published maps and institutional affiliations.

## Supplementary Material

Supplementary Data

## Figures and Tables

**Table 1:** Number of patients and samples used during this study.

		Number of samples	Number of patients
Normal	Ovary	15	13
	Fallopian tube	6	6
Borderline		11	11
Malignant	Serous	65	65
	Endometrioid	7	7
	Clear cell	6	5
