# Supplementary material for: Epithelial ovarian carcinoma diagnosis by desorption electrospray ionization mass spectrometry imaging
Source: Sci Rep. 2016 Dec 15;6:39219. doi: 10.1038/srep39219 (PMC5156945; doi:10.1038/srep39219)
Supplement: Supplementary Data [file srep39219-s1.pdf]

Supplementary Data

Epithelial ovarian carcinoma diagnosis by Desorption Electrospray Ionization Mass Spectrometry Imaging

Maria Luisa Dória, James S. McKenzie, Anna Mróz, David L Phelps, Abigail Speller, Francesca Rosini, Nicole Strittmatter, Ottmar Golf, Kirill Veselkov, Robert Brown, Sadaf Ghaem-Maghami, Zoltan Takats

Department of Surgery and Cancer, Faculty of Medicine, Imperial College London, London, United Kingdom

a)

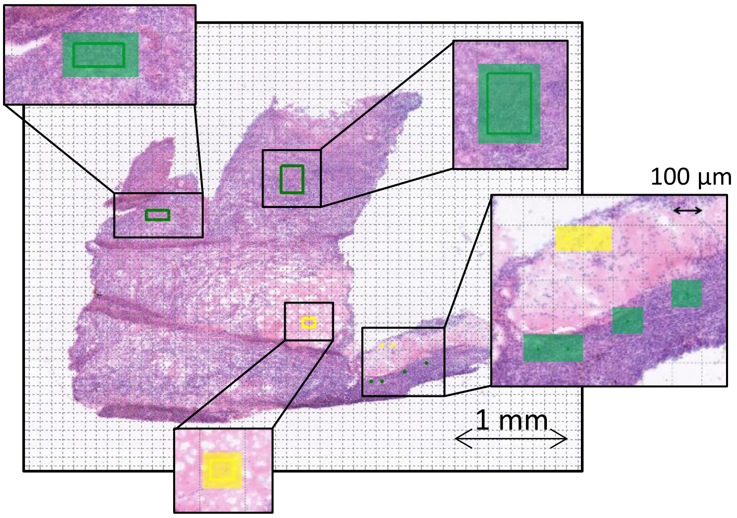

b)

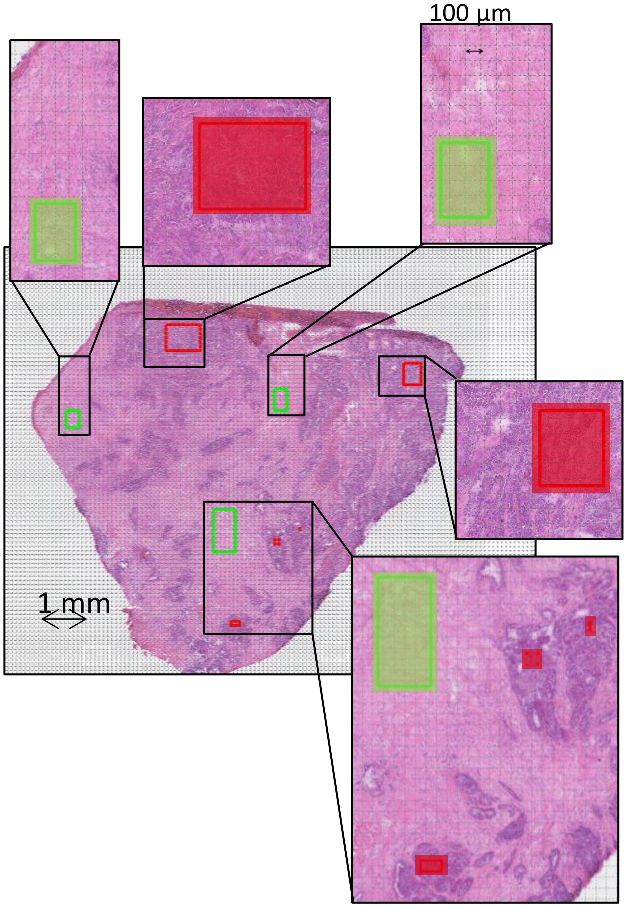

Supplementary Figure 1: Detailed annotations in a) Normal ovary with two different tissue types, normal stroma (green) and corpus albicans (yellow).and b) Serous carcinoma with two tissue types in each sample, stroma (green) and tumour tissue (red) Each grade square correspond to one pixel / spectrum.

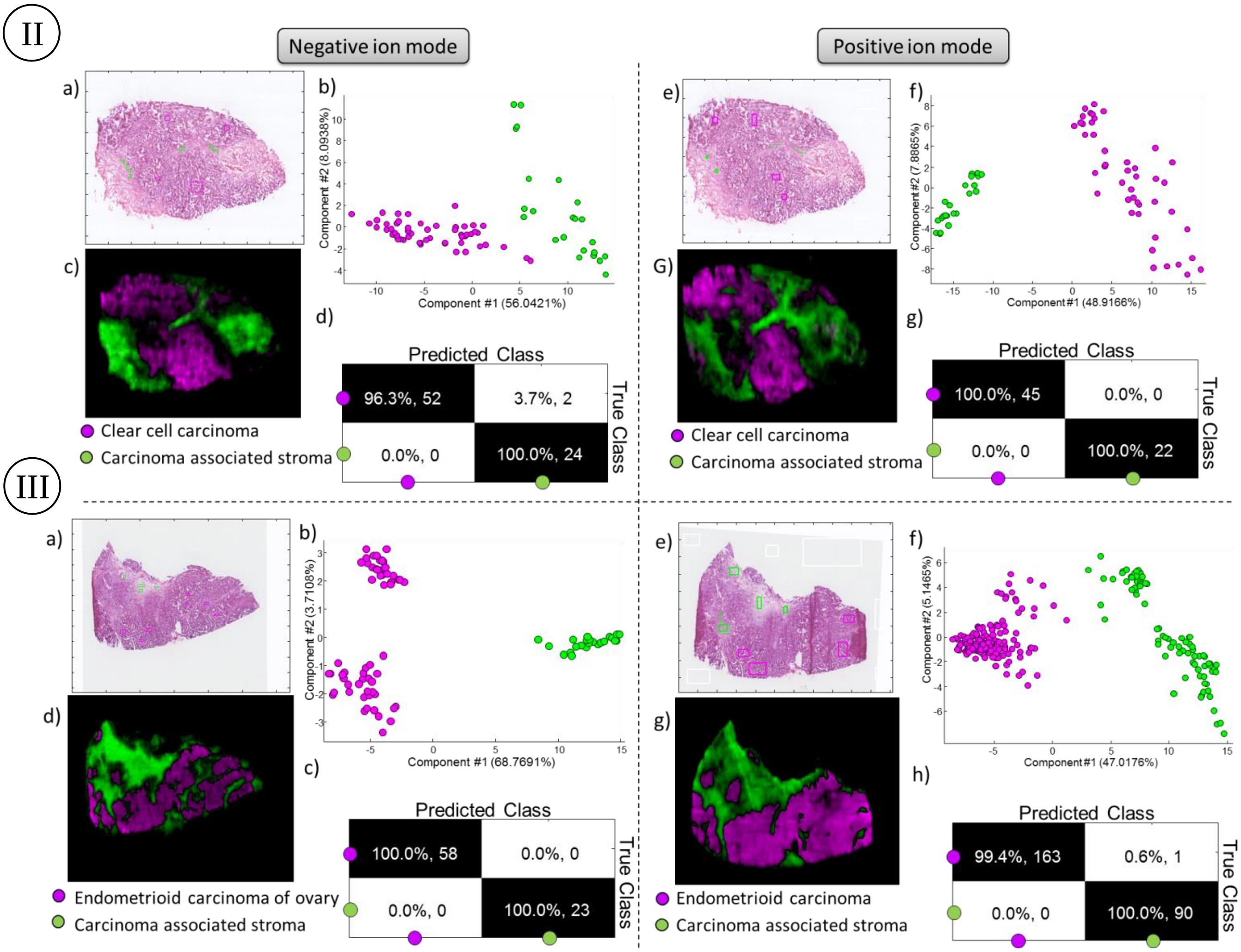

Supplementary Figure 2: Two different carcinomas analysed in negative and positive ion mode, I clear cell carcinoma II and endometrioid carcinoma. Each sample has two tissue types, stroma (green) and carcinoma tissue (pink), Data acquired in negative ion mode and positive ion mode. a) and e) Optical image and b) and f) PCA analysis of selected regions of interest. c) and g) predicted RMMC components with d) and h) leave one out cross validation together

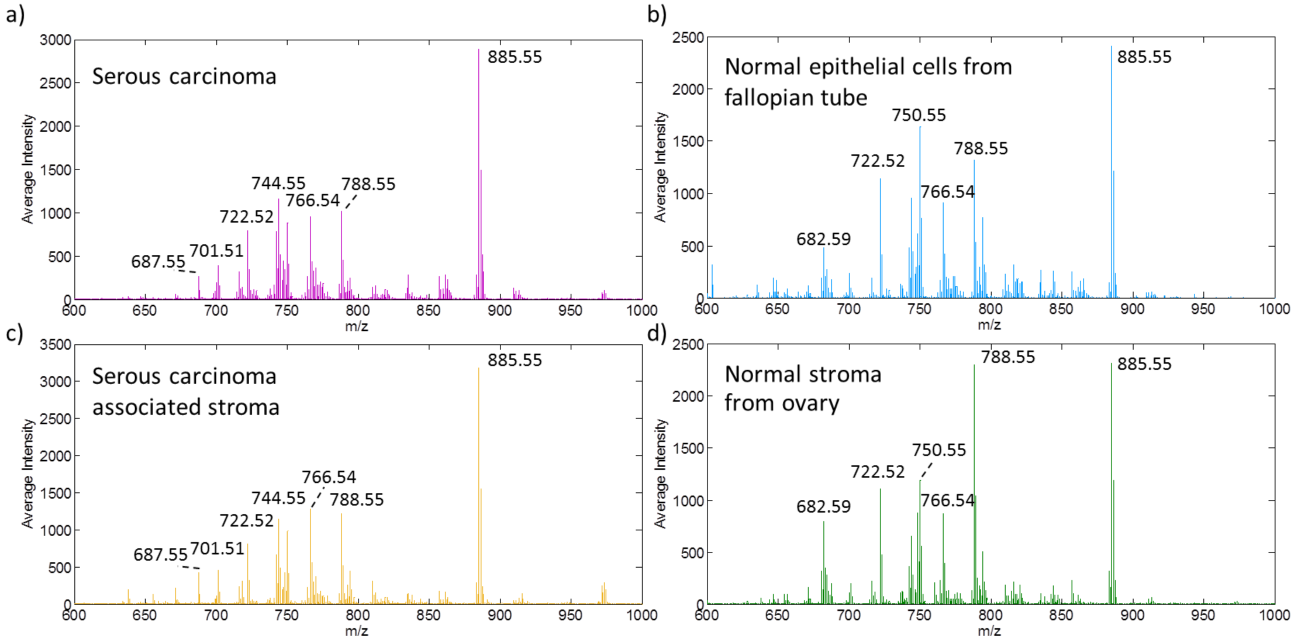

Supplementary Figure 3: Average spectra of four different tissue types, a) Serous carcinoma from ovary, b) normal epithelium from healthy fallopian tube, c) Serous carcinoma associated stroma and d) normal stroma from normal appearance ovary.

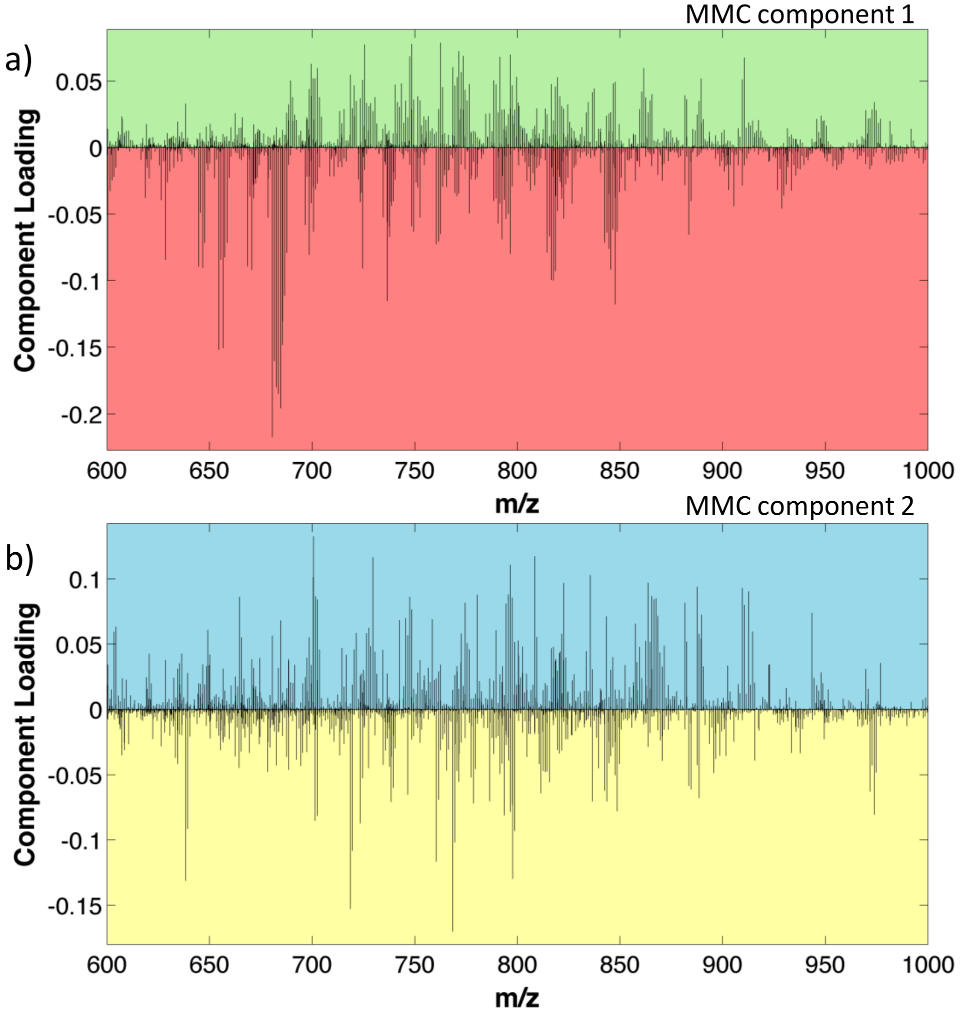

Supplementary Figure 4: RMMC component weights for latent variables a) 1 and b) 2.

m/z	adduct	ID	Loading Value
725.5164	$[M-H]^-$	PA(38:3)	0.08
762.5125	$[M-H]^-$	PE(38:6)	0.08
771.5225	$[M-H]^-$	PG(36:3)	0.07
796.5915	$[M-H]^-$	PE(40:3)	0.07
654.5625	$[M+Cl]^-$	Cer(d40:2)	-0.15
656.5785	$[M+Cl]^-$	Cer(d40:1)	-0.15
684.5925	$[M+Cl]^-$	Cer(d42:1)	-0.2

m/z	adduct	ID	Loading Value
796.5465	$[M-H]^-$	PS(P-38:3)/ PS(O-38:4)	0.11
808.5056	$[M-H]^-$	PS(38:5)	0.12
718.5425	$[M-H]^-$	PE(34:0)	-0.15
760.5175	$[M-H]^-$	PS(34:1)	-0.12

Positive latent variable 1
Negative latent variable 1
Positive latent variable 2
Negative latent variable 2

Supplementary Table 1: Identified *m/z* values with the highest loading values from RMMC component weights 1 and 2

Supplementary Table 2: *m/z* values with possible IDs, p-values, q-values and class where they are significant, together with mean intensities and mean log_2 fold change equal or greater than 1

					MEAN				MEAN Fold Change		
m/z	**IDs**	**Tissue Diff**	**ANOVA pVal**	**ANOVA qVal**	**HealthyFT**	**HealthyOv**	**Serous**	**Stroma**	**Serous/ HealthyOv**	**Serous/ Stroma**	**Stroma/ HealthyOv**
701.52	PA(36:1)	Stroma	6.48E-06	1.28E-04	92.2277	198.6975	392.0609	454.1372	1.0	-0.2	1.2
723.50	PA(38:4)	Stroma	1.38E-04	1.68E-03	5.8757	23.0745	47.5043	74.0624	1.0	-0.6	1.7
699.50	PA(36:2)	Serous	2.16E-06	4.71E-05	18.6893	24.2049	96.1852	68.2801	2.0	0.5	1.5
725.52	PA(38:3)	Serous	1.33E-03	1.29E-02	7.7482	7.2642	61.7486	54.1963	3.1	0.2	2.9
747.50	PA(40:6)	Serous	1.27E-04	1.57E-03	0	1.0101	38.202	28.5214	5.2	0.4	4.8
760.52	PS(34:1)	HealthyOv	1.11E-16	1.55E-14	4.3843	206.877	45.8586	44.7503	-2.2	0.0	-2.2
842.60	PS(40:2)	HealthyOv	9.63E-11	5.01E-09	17.5186	88.463	19.6866	23.9334	-2.2	-0.3	-1.9
796.55	PS(P-38:3)	HealthyFT	2.23E-308	3.89E-306	245.536	131.3943	47.2799	32.8963	-1.5	0.5	-2.0
822.56	PS(P-40:4)	HealthyOv	3.62E-06	7.57E-05	33.8631	37.169	15.5539	8.5823	-1.3	0.9	-2.1
788.55	PS(36:1)	HealthyOv	2.23E-308	4.18E-306	1320.4233	2302.4566	1013.3137	1217.6882	-1.2	-0.3	-0.9
836.55	PS(40:5)	Stroma	1.10E-02	1.00E+00	29.6201	17.3117	41.288	61.5108	1.3	-0.6	1.8
834.53	PS(40:6)	HealthyFT	1.21E-02	1.00E+00	140.6539	33.1473	131.7306	130.4371	2.0	0.0	2.0
862.56	PS(42:6)	Serous	1.21E-08	4.30E-07	58.367	29.0567	126.6541	59.8317	2.1	1.1	1.0
860.54	PS(42:7)	Serous	3.22E-08	1.05E-06	13.01	12.3084	55.6455	21.8686	2.2	1.3	0.8
888.57	PS(44:7)	Serous	4.14E-09	1.58E-07	123.359	68.0586	320.5055	134.6557	2.2	1.3	1.0
864.57	PS(42:5)	Serous	2.15E-06	4.72E-05	52.4406	14.7539	104.446	34.5072	2.8	1.6	1.2
836.54	PS(40:5)	Serous	3.29E-04	3.60E-03	13.4375	4.7497	56.7163	13.9188	3.6	2.0	1.6
882.53	PS(44:10)	Serous	3.53E-06	7.40E-05	1.412	0.9965	20.257	3.815	4.3	2.4	1.9
748.53	PE(P-38:5)	HealthyOv	9.93E-13	7.14E-11	615.4524	879.678	344.2639	418.3954	-1.4	-0.3	-1.1
714.51	PE(34:2)	Serous	3.42E-06	7.21E-05	14.9338	41.8857	85.5191	35.2683	1.0	1.3	-0.2
742.54	PE(36:2)	Serous	1.77E-06	3.99E-05	477.5988	366.1549	786.0731	660.3064	1.1	0.3	0.9
768.56	PE(38:3)	Serous	1.58E-06	3.61E-05	196.3088	126.3147	329.4129	293.3299	1.4	0.2	1.2
726.55	PE(P-36:2)	Serous	2.00E-06	4.44E-05	85.9153	41.6245	109.5308	41.062	1.4	1.4	0.0
740.53	PE(36:3)	Serous	1.20E-06	2.82E-05	52.5928	59.9218	162.1856	85.3712	1.4	0.9	0.5
770.57	PE(38:2)	Stroma	1.50E-06	3.47E-05	180.8012	132.4103	370.0506	375.8993	1.5	0.0	1.5
690.51	PE(32:0)	Serous	3.14E-03	2.78E-02	1.6379	8.2168	26.3467	16.7422	1.7	0.7	1.0
728.56	PE(P-36:1)	Serous	2.47E-07	6.68E-06	72.7844	31.037	103.3221	40.8148	1.7	1.3	0.4
724.53	PE(P-36:3)	Serous	1.39E-07	3.88E-06	11.8338	30.1446	102.4665	43.2861	1.8	1.2	0.5
772.59	PE(38:1)	Serous	7.40E-05	9.96E-04	82.7213	47.5517	168.408	126.2506	1.8	0.4	1.4
752.56	PE(P-38:3)	Serous	2.01E-04	2.33E-03	9.4364	21.0165	80.2659	47.0134	1.9	0.8	1.2
820.59	PE(42:5)	Serous	7.73E-04	7.77E-03	0	3.9672	15.942	9.6812	2.0	0.7	1.3
788.53	PE(40:7)	Serous	2.06E-06	4.53E-05	20.052	13.0912	59.6556	32.1204	2.2	0.9	1.3
698.52	PE(P-34:2)	Serous	4.27E-05	6.38E-04	13.8066	12.4434	68.5407	14.4298	2.5	2.2	0.2
818.58	PE(42:6)	Serous	2.49E-04	2.81E-03	0	2.3533	17.885	10.3796	2.9	0.8	2.1
720.50	PE(P-36:5)	Serous	5.61E-05	7.93E-04	9.8902	3.7886	31.5205	8.1097	3.1	2.0	1.1
796.59	PE(40:3)	Serous	5.08E-06	1.03E-04	5.9128	3.9787	38.4611	24.3157	3.3	0.7	2.6
816.56	PE(42:7)	Serous	5.24E-10	2.33E-08	0	1.3278	14.9384	5.1635	3.5	1.5	2.0
762.51	PE(38:6)	Serous	5.92E-05	8.27E-04	48.5122	4.8984	80.8939	49.1363	4.0	0.7	3.3
819.53	PI(P-34:1)	HealthyOv	4.35E-05	6.49E-04	0	41.7619	6.4232	3.1178	-2.7	1.0	-3.7
847.57	PI(P-36:1)	HealthyOv	4.13E-07	1.08E-05	5.0527	15.8385	3.5778	3.0651	-2.1	0.2	-2.4
911.57	PI(40:5)	Serous	2.53E-07	6.81E-06	47.3651	46.3707	98.5977	51.0697	1.1	0.9	0.1
835.54	PI(34:1) (TG2)	Serous	1.73E-06	3.92E-05	259.8877	103.4147	285.9541	149.766	1.5	0.9	0.5
861.55	PI(36:2) (TG2)	Serous	2.14E-08	7.07E-07	101.657	84.4465	283.8352	163.8657	1.7	0.8	1.0
833.52	PI(34:2) (TG2)	Serous	2.31E-06	5.00E-05	33.1697	21.5355	74.2741	27.9	1.8	1.4	0.4
859.54	PI(36:3)	Serous	4.75E-08	1.47E-06	68.0294	29.795	124.4219	56.1878	2.1	1.1	0.9
809.52	PI(32:0)	Serous	1.51E-02	1.00E+00	0.8766	5.2895	25.2308	8.1522	2.3	1.6	0.6
863.57	PI(36:1)/TG(P-56:19)	Serous	2.45E-06	5.28E-05	163.2614	44.8395	227.9436	89.0283	2.3	1.4	1.0
881.52	PI(38:6)	Serous	5.55E-07	1.40E-05	14.5945	7.0277	50.9248	13.0963	2.9	2.0	0.9
909.55	PI(40:6)	Serous	9.92E-12	5.92E-10	46.864	17.8414	131.5908	44.6693	2.9	1.6	1.3
889.57	PI(38:2)/TG(P-58:20)	Serous	4.49E-07	1.16E-05	2.6187	5.9681	55.4325	21.448	3.2	1.4	1.8
865.57	PI(36:0)	Serous	1.06E-05	1.98E-04	1.6403	0.3586	18.751	4.0434	5.7	2.2	3.5
654.56	Cer(d40:2)	HealthyOv	2.23E-308	3.63E-305	50.5737	91.8955	0.2695	0.0816	-8.4	1.7	-10.1
656.58	Cer(d40:1)	HealthyOv	2.23E-308	1.81E-305	86.7238	98.782	1.9053	1.2988	-5.7	0.6	-6.2
682.59	Cer(d42:2)	HealthyOv	2.23E-308	7.77E-306	478.2389	792.2141	25.3537	29.6576	-5.0	-0.2	-4.7

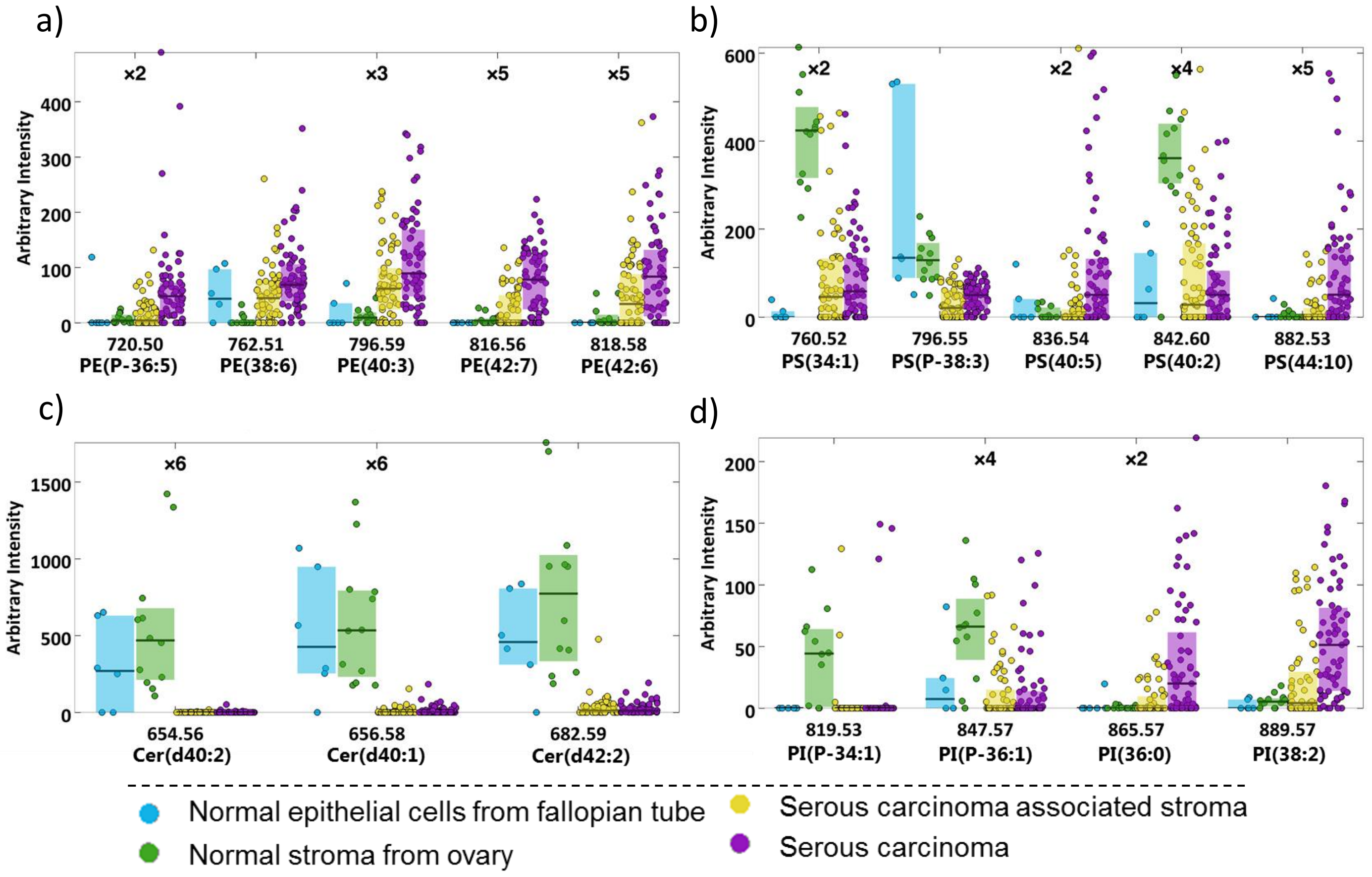

Supplementary Figure 5: Box plots of different lipid species form five phospholipid and sphingolipid classes a) phosphatidylethanolamine, b) phosphatidylserine, c) ceramide and d) phosphatidylinositol

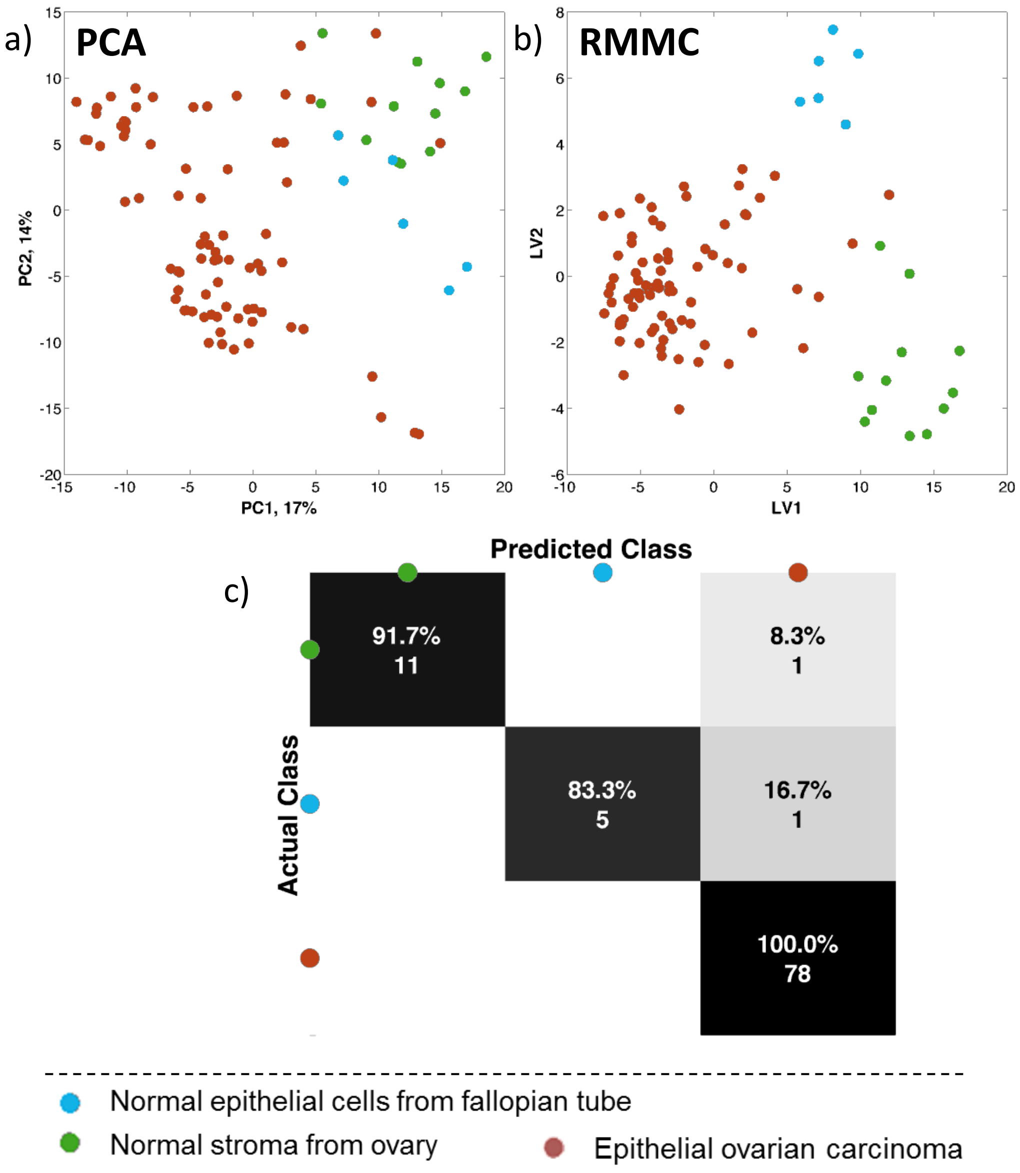

Supplementary Figure 6: Statistical analysis of DESI MS spectrum between *m/z* 600-1000 from all EOCs together, normal stroma from normal ovary and normal epithelium from fallopian tube. a) PCA and b) RMMC scores plots with its respective c) leave-one-patient-out cross validation results

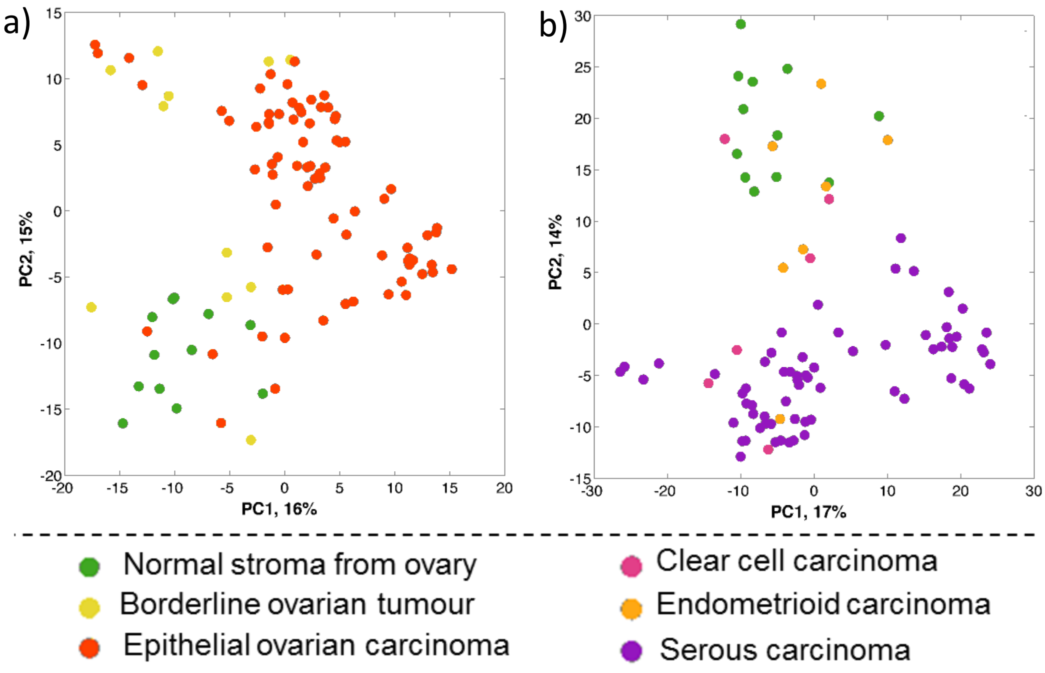

Supplementary Figure 7: Principle component analysis (PCA) of DESI MS spectrum between 600-1000Da from the a) all carcinomas together, borderline ovarian tumours and normal stroma from normal ovary, and b) different carcinomas as Serous, Endometrioid and Clear cell carcinomas together with normal stroma from normal ovary

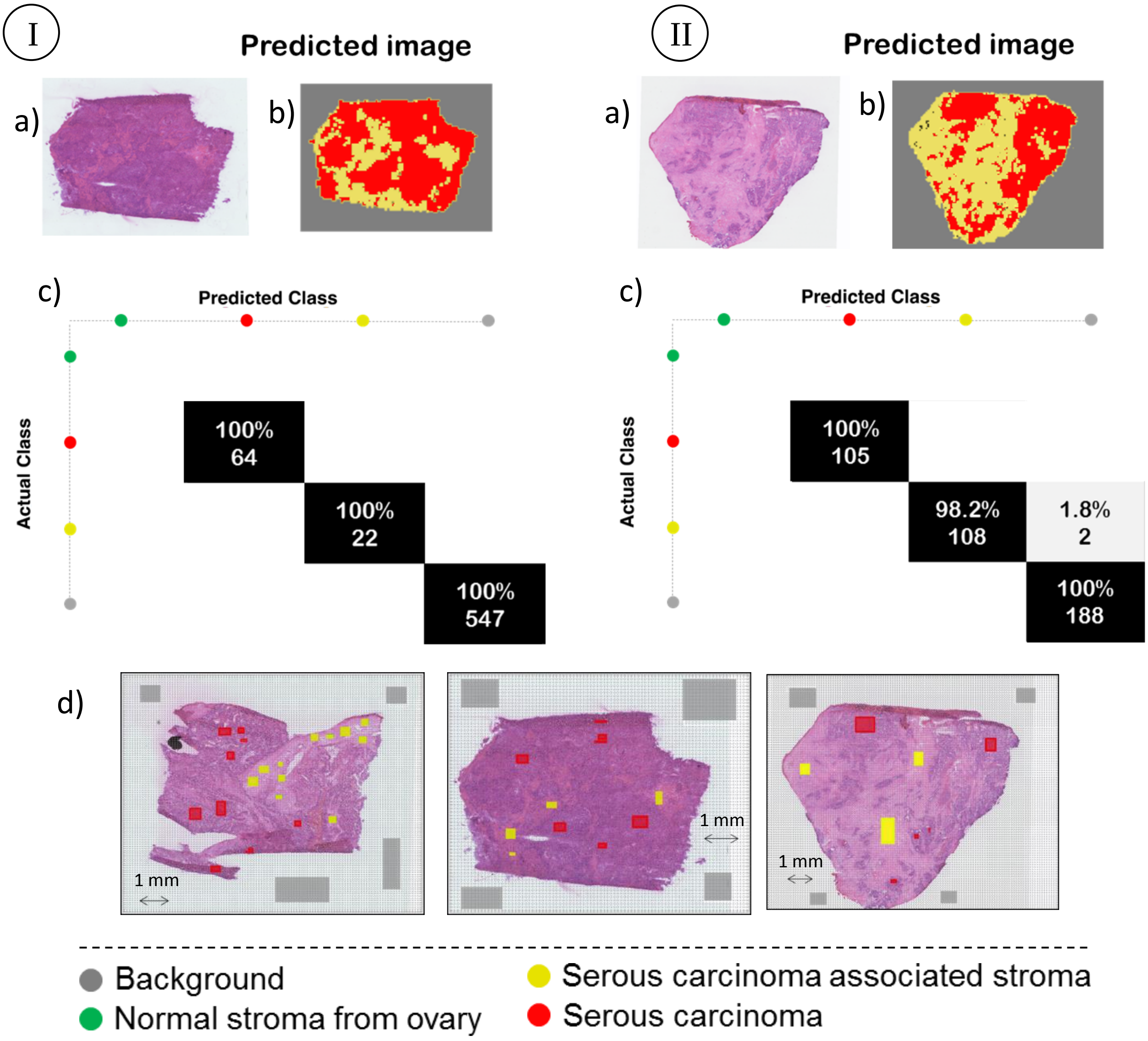

Supplementary Figure 8: Prediction of two independent sample using as training set the previous model (serous ovarian carcinoma). a) Optical image of the independent sample, b) predicted image and c) Leave one out cross validation results and d) respective regions of interest marked by histopathologist used for this validation.
